# Heterotic Effect of Different Cytoplasmic Combinations in Sunflower Hybrids Cultivated Under Diverse Irrigation Regimes

**DOI:** 10.3390/plants9040465

**Published:** 2020-04-07

**Authors:** Vikrant Tyagi, Satwinder Kaur Dhillon, Gurpreet Kaur, Prashant Kaushik

**Affiliations:** 1 Department of Plant Breeding and Genetics, Punjab Agricultural University, Ludhiana 141004, India; vikrant@pau.edu (V.T.); sklb-pbg@pau.edu (S.K.D.); gurpreetkaur@pau.edu (G.K.); 2 Instituto de Conservación y Mejora de la Agrodiversidad Valenciana, Universitat Politècnica de València, 46022 Valencia, Spain; 3 Nagano University, 1088 Komaki, Ueda, 386-0031 Nagano, Japan

**Keywords:** *Helianthus annuus*, heterosis, water stress, cytoplasmic male sterility, hybrids

## Abstract

The sunflower hybrids hold a narrow cytoplasmic diversity. Besides, the heterotic effect of wild cytoplasmic combinations of sunflower on important traits under water stress has not been explored in detail. Here, we evaluated the different sunflower cytoplasmic combinations in sunflower hybrids using cytoplasmic male sterile (CMS) sources as female parents. We used a total of sixteen sunflower genotypes representing twelve CMS lines from wild and conventional sources along with four restorer lines. Twelve CMS lines were crossed with four restorer lines to develop a total of 48 F_1_ hybrid combinations. The hybrids were evaluated under two different environments (i.e., regular irrigation and water stress) for morphophysiological, yield, and biochemical traits over two years. Heterotic effect for various CMS sources was evaluated on all of the three possible scales, namely, better-parent heterosis (BPH), mid-parent heterosis (MPH), and heterosis as percent of check (PSH-996). For better-parent and mid-parent heterosis, the CMS sources *Helianthus annuus, Helianthus argophyllus*, and *Helianthus debilis* demonstrated positive better-parent heterosis for seed yield, oil content, and oleic acid irrespective of the environment. However, the hybrid combinations of different sources when using the genotype RCR8297 as the restorer parent recorded maximum average returns. Furthermore, chlorophyll meter (SPAD) reading positively correlated with days to 50% flowering, days to maturity, plant height, and number of leaves per plant in both the environments. Overall, this study identified and compared the heterotic effect of the different cytoplasmic combinations in sunflower under water stress as well as under normal irrigation environments.

## 1. Introduction

Sunflower (*Helianthus annuus* L.) is an important oilseed crop and sunflower oil is visualised as the most potential oil to narrow the gap between the total requirements and the domestic production of edible oil in India [1]. Sunflower is widely adopted and valued for its high-quality edible oil due to the presence of polyunsaturated fatty acids in conjunction with the right amount of linoleic acid and oleic acid, which are known to reduce the risk of cardiac problems [2,3]. Sunflower hybrids are preferred over varietal populations as hybrids offer several benefits in terms of growth, development, synchronous flowering, early maturity, higher seed setting, increased productivity, fewer harvest losses, and uniform seed moisture content for storage purposes [4]. Therefore, breeding efforts in sunflower are more focused on exploitation of heterosis, which has been established as a useful tool where genetically divergent parents result in highly productive sunflower hybrids with agronomically superior traits [5]. Genetic divergence among parents of a hybrid combination is a prerequisite for hybrid superiority, thus making genetic diversity evaluation an essential component of plant breeding programs [6]. The information on genetic relatedness among parents increases the selection efficiency of parents and reduces the chances of selecting the genotypes of similar genetic composition [7,8]. Therefore, in a systematic breeding program, it is essential to identify superior parents for hybridization and crosses to expand the genetic variability for selection of superior genotypes [9].

Water stress is one of the significant causes for crop losses worldwide, reducing average yields to 50 percent or even more; furthermore, possible global climate change scenario suggests a future upsurge in drought stress [10,11]. Therefore, breeding crop varieties for improved water use efficiency is of utmost importance [12]. Sunflower, being a highly cross-pollinated crop, is ideally suited for the exploitation of heterosis. A breeding program generally aims at developing cultivars with high grain yield and oil yield potential [13]. Consequently, there have been relatively fewer efforts to diversify the inbreds to get better heterosis over check hybrids. The development of sterile cytoplasmic male sterility (CMS) analogs of lines used in sunflower breeding programs for commercial hybrid development is one of the practical applications of CMS investigations [14]. At present, only one CMS source (i.e., PET-1) is being widely used for sunflower hybrid breeding program [15]. This cytoplasmic uniformity poses a potential risk for hybrid sunflower production [16]. The utilization of different cytoplasmic backgrounds in hybrid development will improve the general variability of sunflower plants and lessen the threats of epiphytotic growing needs for additional genetic variability to improve cultivated sunflower plants [17].

Keeping in view the importance of diversifying the CMS sources in sunflower hybrids and exploiting heterosis under normal as well as under stress conditions, efforts were made to study twelve CMS lines belonging to different cytoplasmic sources and their hybrid combinations with four common restorers for estimation of better-parent heterosis (%), mid-parent heterosis (%), and heterosis over the popular commercial check PSH-996 (%).

## 2. Results

The overall mean performance of parents and their hybrids under normal as well stress conditions is provided in Tables S1 and S2.

### 2.1. Better-Parent Heterosis (BPH)

The average values of BPH pooled over the years are provided in Table S3. There were significant differences concerning BPH for all the traits studied under normal environment as well as under stress environment. However, *Helianthus debilis* showed the maximum negative BPH with all restorers for days to flowering and maturity compared to *Helianthus petiolaris* (a commercial CMS source), which was determined to be suitable for early maturing hybrids in sunflower (Table 1). *Helianthus argophyllus* CMS sources performed well under both the environments for seed yield and oil content in combination with different restorers. The highest significant positive BPH for seed yield was recorded for *H. annuus* sources with the restorer P124R (199.37%) under the regular water regime (Table 1), while under water stress conditions, *H. petiolaris* sources showed higher heterosis over better parent for seed yield with the restorer P69R (115.40%) (Table 2). For oil content, the CMS source *H. debilis* showed negative heterosis over better parent with all four restorers in the regular water regime (Table 1). The highest positive BPH for oil content under water stress was observed for *H. annuus* (15.07%) and *H. argophyllus* (13.64%) sources with the restorer P100R (Table 2). In contrast, under the regular water regime, the CMS sources *H. argophyllus* (7.83%) and *H. annuus* (7.75%) demonstrated high BPH for oil content (Table 1).

Negative values for BPH for fatty acid composition were observed under normal water irrigation, whereas a few positive values were recorded under water stress conditions. For linoleic acid (%), significant positive BPH values were demonstrated for three restorers (except RCR8297) with all CMS sources under normal water irrigation (Table 1). The CMS source *H. debilis* showed maximum heterosis with the restorer P69R for linoleic acid under water stress conditions (Table 2). The CMS sources *H. argophyllus*, *H. annuus*, and *H. debilis* showed positive BPH under both the environments for seed yield and oil content. These sources could be a good option for use in future sunflower breeding programs for the development of water-use-efficient sunflower hybrids. Further, the trends of BPH values for the yield and oil content are provided in Figure S1.

### 2.2. Mid-Parent Heterosis (MPH)

The average values of MPH pooled over the years are provided in Table S4. The highest MPH values for seed yield were observed for *H. annuus* CMS sources in combination with RCR8297 (220.1%), followed by P124R (213.82%), P100R (185.94%), and P69R (156.35%), under normal irrigation conditions (Table 3). The *H. petiolaris* sources recorded the highest significant positive heterosis over mid-parent when crossed with P69R (158.73%) and P100R (106.74%) under the water stress environment (Table 4). For oil content, all CMS sources showed positive MPH values, except the *H. debilis* source with P100R (–3.91%) and P124R (–5.50%), and *H. annuus* with RCR8297 (–1.66%) under the normal water regime (Table 3). All CMS sources’ combination with all four restorers were observed to have positive MPH under water stress conditions (Table 4). The highest MPH for oil content was observed for *H. praecox* with P124R (13.20%) under the normal water regime and the *H. debilis* source with P69R (59.43%) under the water stress environment (Table 3 and Table 4). Except the *H. annuus* CMS sources in combination with the pollen parent RCR8297 (–2.73%), all other CMS sources showed a positive MPH for linoleic acid (%) under the normal water regime; and CMS sources *H. argophyllus* with P124R (–2.33%), and *H. petiolaris* (–3.92%) and *H. praecox* (–6.10%) with RCR8297 showed negative MPH, while the remaining combinations had positive MPH under the water stress regime (Table 4 and Table 5). The *H. petiolaris* CMS sources had higher MPH values with three restorers, except P124R, for head diameter and *H. annuus* sources had higher MPH values for plant height under both the water regimes (Table 3 and Table 4). All of the CMS sources had positive MPH values for seed yield. For oil content, the CMS sources *H. debilis* and *H. annuus* showed negative MPH under normal conditions. Further, the trends of MPH values for yield and oil content are provided in Figure S2.

### 2.3. Heterosis as Percent of Check

The average values of heterosis over the commercial check pooled over the years are provided in Table S3. The average performance of sunflower hybrids (% of the check) developed across groups is presented in Table 5 and Table 6. The highest average performance for seed yield with respect to check was observed for the *H. debilis* source with RCR8297 (120.86%) followed by *H. argophyllus* with RCR8297 (111.04%) under the normal irrigation regime (Table 5). In the water stress regime, the *H. debilis* source with P100R (138.30%) and RCR8297 (138.24%), and *H. annuus* with RCR8297 (136.55%) showed significant and positive heterotic effects (Table 6). All the CMS sources with all four restorers were observed to have positive values for oil content under the water stress environment (Table 6). The male parent RCR8297 was recorded as the best performer under water stress as well as normal irrigation conditions for seed yield (Table 5). For seed yield, the CMS sources *H. argophyllus*, *H. annuus, H. debilis*, and *H. petiolaris* combinations with P100R; *H. petiolaris* with P69R; and *H. argophyllus*, *H. debilis*, *H. petiolaris*, and *H. praecox* with RCR8297 recorded significant heterosis under normal water irrigation (Table 5 and Table 6). For harvest index under normal conditions, *H. praecox* was identified as having the highest heterosis over commercial check, whereas *H. debilis* was identified as having the highest heterosis for harvest index under the water stress environment (Table 5 and Table 6). For oil content, *H. argophyllus* and *H. debilis* were recorded as having the highest heterosis over commercial check under the water stress environment and normal environment, respectively (Table 5 and Table 6). Further, the trends of heterosis percent of check values for yield and oil content are provided in Figure S3.

### 2.4. Correlations

For parental lines and hybrids grown under the regular water regime, 21 correlation coefficients were found to be significant (*p* < 0.05) (Table 7). Among these, five were negative correlations. Under normal conditions, plant height was correlated with the number of leaves per plant, oleic acid content, days to maturity, SPAD reading, and seed per plant (Table 7), whereas 100-seed weight was correlated with plant height, number of leaves per plant, and oleic acid content (Table 7).

Likewise, under the water stress environment, only 11 correlation coefficients were determined to be significant (*p* < 0.05) (Table 8). Furthermore, there was no significantly negative correlation recorded under water stress, and the correlation values were not significant for most of the traits (Table 8). SPAD reading was positively associated with days to 50% flowering, days to maturity, plant height, and number of leaves per plant (Table 8). Significant correlations were determined between plant height and number of leaves per plant and also between days to maturity and days to 50% flowering (Table 8).

## 3. Discussion

Sunflower is a highly cross-pollinated crop and commercial cultivation of its hybrids is more desired because of their agronomic and economic advantages over varieties (high productivity, high oil content, uniformity, etc.) [18]. Moreover, the favorable characteristics of sunflower hybrids like production stability, response to high-input agriculture, high self-fertility, consistent growth, and maturity shifted the focus toward heterosis breeding, leading to the release of first-ever sunflower hybrid BSH-1 in India, which provided the required fillip to expand sunflower cultivation in the country [19]. Since then, many hybrids have been released for commercial cultivation based on the cytoplasmic genetic male sterility system. The central component of sunflower hybrid development is cytoplasmic male sterility. The synthesis of hybrids with high heterotic effect became possible after the discovery of the first CMS source by Leclercq [20].

The range of heterosis in sunflower is highly variable for different agronomic and yield traits, especially for seed yield. Significant positive heterosis for this trait has been reported by several researchers [17,21,22,23]. The wild relatives of crop plants were evolved in wild environments independent of the pressure by human selection [24]. Therefore, they are the storehouse of valuable genes for traits like drought resistance, and employing the useful genes from wild relatives of the crop has resulted in the development of drought-tolerant genotypes in several crops like maize and eggplant [25,26,27,28]. Similarly, the wild relatives of sunflower were used in the past [29,30,31].

Here, we evaluated the agronomical and biochemical traits in the hybrids resulting from a cross of 12 CMS lines with 4 restorer lines as male parents. These dwarf hybrids are probably suitable for mechanization and intercropping without much reduction in their yield levels [32]. In this work, the CMS source *H. argophyllus* was observed to have a negative heterotic effect, which is suitable for dwarf plant-type hybrid. The results indicated a positive influence of cytoplasmic sources on heterosis for head diameter, particularly under the water stress environment, which should be exploited to develop high-yielding hybrids suitable for growing under moisture stress environments [5]. Seed yield is one of the most critical traits for sunflower breeders. The evaluation for the essential traits in sunflower hybrids should start from inbred line selection by estimating the heterosis in the hybrid combinations and also by determining the correlation among the most important characteristics to develop a hybrid with a combination of desired traits [33]. Sunflower has been proposed as a model oilseed for changing climate needs. Therefore, more focus is needed in achieving stable yield under water stress.

Moreover, hybrids with short vegetative cycles are also desirable for popularizing sunflower cultivation in non-traditional sunflower-growing regions [34]. Crop domestication has resulted in less genetic diversity than that of the species as a whole. This narrowing of the genetic base of cultivated sunflower is causing a problem in the successful production of sunflower [35]. The introgression from the wild relatives of sunflower is useful in widening the genetic basis of sunflower. The wild relatives of sunflower represent the vital source of CMS for cultivated sunflower plants. The wild relatives of sunflower continue to contribute to sunflower improvement, and there is still a lot of potential to be exploited [36]. Similarly, in this work, the wild relatives of sunflower when used as the CMS sources, especially *H. argophyllus* and *H. debilis*, demonstrated positive better parent heterosis for seed yield, oil content, and oleic acid irrespective of the environment.

The overall significant differences in the performance of different wild sources have been observed [37]. It was readily apparent that various cytoplasmic sources influenced traits under both the environments under consideration. Similarly, previous studies in sunflower have proved that the wild cytoplasmic sources significantly affect the qualitative and quantitative traits [38]. In this extensive study, the cytoplasmic effect on heterosis along with water stress tolerance was identified using the different wild species. CMS sources significantly influencing the hybrid vigour under the moisture stress environment were identified. Further, the sources belonging to *H. argophyllus*, *H. annuus*, and *H. debilis* were determined to be comparable with the commercially used CMS source PET-1 and were at par in BPH for seed and oil content under stress conditions.

## 4. Materials and Methods

### 4.1. Experimental Material

The study was carried out under open field conditions at the Punjab Agricultural University (PAU), Ludhiana, India (coordinates: 30°54′6′′N, 75°48′27′′E). The experimental material consisted of 48 F_1_ hybrids with different CMS backgrounds, sixteen parental lines, and one commercial check hybrid. The hybrids were produced by crossing eight alloplasmic CMS lines having cytoplasm from various wild sources and four CMS lines from a cultivated source (cytoplasm from *H. petiolaris*) with four restorer lines (Table 9). The experiment was conducted over two years over the two environments (normal irrigated and water stress). Water stress was created by withholding the irrigation throughout anthesis and soft dough phases of crop growth [39]. In the normal irrigation environment, six irrigations (recommended by PAU for cultivation of sunflower in the Punjab region) were applied during the crop season, whereas in the water stress environment, only two irrigations were applied during the crop season to create water stress for both years.

A set of 48 male sterile line (A) × male fertile line (R) crosses and parental lines along with PSH-996 (a check hybrid based on the PET-1 source released by the Punjab Agricultural University, Punjab, India) were planted during spring 2011 in the first week of February in a randomized block design (RBD) with three replications. Each genotype was represented by a plot of two rows of 3 m length each. The crosses evaluated in spring season 2011 were again synthesized in offseason 2011 for second-year evaluation in spring 2012. The same set of experiment conducted in spring 2011 was repeated in spring 2012. The inter-row and intra-row spacing were maintained at 60 cm and 30 cm, respectively, during both the years of the experiment. All plant production practices were followed as defined elsewhere, and no phytosanitary measures were needed. Total amount of rainfall for each season (i.e., 316.7 and 42.2 mm for year 1 and 2, respectively) and data of rainfall (mm) recorded during the first and second year of the experiment are provided in Table S6. 

### 4.2. Morphophysiological Traits

Hybrids showed uniformity for their respective phenotypes and the parental genotypes being inbred lines were also uniform; therefore, five random plants were chosen from each entry in each replication for the estimation of plant traits. Days to 50% flowering were recorded from the date of sowing until approximately 50% of the flower buds opened its flowers in each genotype in all replications. Days to maturity were counted from sowing to full maturity when the backside of the heads turned brown. The data for all other morphological and physiological traits were recorded on the same set of five plants chosen from each genotype. Plant height (cm) was measured in centimetres from ground level to the attachment of head at the time of physiological maturity. Head diameter (cm) was measured from one end of the head to the other end at maturity. The number of leaves per plant were determined by counting the number of leaves in five randomly selected plants in each replication at the time of flowering. Specific leaf weight (SLW) was calculated as the ratio of the dry weight of total leaves per plant (g) to the total number of leaves per plant. Relative leaf water content (RLWC) was determined from 100 mg leaf discs (fresh weight), submerged in distilled water in test tubes until saturation. After 6 h, the leaf discs were removed from test tubes. Surface water of the discs was blotted off without putting any pressure, and then the discs were weighed to obtain saturated weight. After that, by drying the discs at 70 °C for 72 h, their dry weight was determined [40]. Chlorophyll content was recorded using SPAD in five intact plants (third-fourth leaf from the top of the plant) for all genotypes in each replication.

### 4.3. Yield and Biochemical Traits

Sunflower is a large-seeded crop; hence, 100-seed weight was determined instead of 1000-seed weight as in the previous studies [41,42,43,44,45]. Hundred-seed weight was recorded from 100 seeds counted from a random sample of open-pollinated seeds from each genotype in each replication. Seed yield per plant was recorded from five open-pollinated plants in each replication, and then the average was calculated. Harvest index (percent), defined as the ratio of seed yield (SY) to the total biomass (vegetative mass (VM) + Seed Yield) at maturity was calculated as follows:

(1) $$\mathrm{HI}=\frac{Seed\;yield}{Total\;above-ground\;biomass}\times 100$$

#### 4.3.1. Oil Content

To determine oil percent in seeds, a wide-line nuclear magnetic resonance (NMR) instrument (Newport Analyzer MK III-A, Newport Instruments Ltd., Milton Keynes, England) was used. The NMR was standardized by the use of 4 g seed of known oil content. Clean seed samples were first dried for 3 h in an oven at 11 °C. A representative sample (2 g) was used for estimating oil content. The instrument was operated by keeping the calibrations described in [46].

#### 4.3.2. Analysis of Fatty Acid Ethyl Esters (FAEEs)

Gas–liquid chromatography was used for fatty acid estimation. Fatty acids were first converted to their ethyl esters by the standard method of transesterification and the percentage of palmitic acid, stearic acid, oleic acid, and linoleic acid was determined [47]. Briefly, a gas chromatograph fitted with a fused silica capillary column and a flame ionization detector (FID) was used for the separation of esters (Varian CP 3800, USA). A CP-SIL 88-coated column was used as the stationary phase, whereas an unreactive gas such as nitrogen was used as the mobile phase. The FAEE sample (2 μL) was injected to the front and middle injector (FID 1177) of the graph chromatograph. The temperature was set at 200 °C, while the temperatures of the injector and FID were maintained at 230 °C and 250 °C, respectively. Samples were maintained at a constant temperature and pressure. Peak identity of fatty acids is confirmed by a reference standard from Sigma Standard, which was run at the same conditions. The composition of fatty acids was estimated based on the peak area and expressed as the percentage of fatty acids.

### 4.4. Data Analysis

The replicated mean values pooled across years for morphophysiological, yield, and biochemical traits were computed for better-parent heterosis (BPH) and mid-parent heterosis (MPH) using the INDOSTAT software (version 7.5). Commercial check hybrid values were used to calculate the heterosis as percent of check (PSH-996). The pooled-over heterosis values of each source based on the species were used to highlight the species-level differences. Heterosis was calculated using the following formulas:

(2) $$\mathrm{Better}-\mathrm{parent}\;\mathrm{heterosis}\;(\mathrm{BPH})=\frac{\bar{F1}-\bar{\mathrm{BP}}}{\bar{\mathrm{BP}}}\times 100$$

where

$\bar{F1}$ = mean value of F_1_

$\bar{\mathrm{BP}}$ = mean value of better-parent

(3) $$\mathrm{Mid}-\mathrm{parent}\;\mathrm{heterosis}\;(\mathrm{MPH})=\frac{\bar{F1}-\bar{\mathrm{MP}}}{\bar{\mathrm{MP}}}\times 100$$

where

$\bar{F1}$ = mean value of F_1_

$\bar{\mathrm{MP}}$ = mean value of mid-parent.

(4) $$\mathrm{As}\;\mathrm{Percent}\;\mathrm{Check}=\frac{\bar{F1}}{\bar{\mathrm{SC}}}\times 100$$

where

$\bar{F1}$ = mean value of F_1_

$\bar{\mathrm{SC}}$ = mean value of standard check hybrid.

The test of significance for BPH, MPH, and over commercial check PSH-996 was determined with the CD (critical difference) value, which was calculated by multiplying SD_d_ with t-value (at both error df *p* ≤ 0.05 and *p* ≤ 0.01 level of significance),

where

SD_d_ = ± $\sqrt{2\mathrm{MSE}/r}\sqrt{2\mathrm{MSE}/r}$

MSE = error mean square as calculated in RBD using parents, F_1_ hybrids, and

standard checks

r = number of replications.

The R package corrplot was used to determine and plot the Pearson’s linear correlation coefficients [48].

## 5. Conclusions

Heterosis has been exploited extensively in crop production and has been a dominant force in the evolution of plants. In sunflower, the hybrids are preferred for their better yield and contributing traits along with their better performance under stresses. In our study, by using wild cytoplasmic sources, we demonstrated that the wild/non-conventional cytoplasmic sources of sunflower had a significant influence on different traits compared to the conventional source PET-1 under normal as well as water stress conditions. Significant differences between MPH and BPH percentages were observed under both situations. The wild CMS sources *H. argophyllus*, *H. debilis*, and *H. praecox*, performed well under both the environments compared to the PET-1 source for seed yield and oil content. High-yielding and water-stress-tolerant hybrids can be developed by using these CMS sources in sunflower heterosis breeding programs.

## Figures and Tables

**Table 1:** Heterosis over better-parent values (BPH, %) among cytoplasmic male sterile (CMS) sources representing five different species of sunflower for morphophysiological, yield, and biochemical traits under normal irrigation conditions.

Traits	P100R					P124R					P69R					RCR8297				
	^+^HA	HAN	HD	HPE	HPR	HA	HAN	HD	HPE	HPR	HA	HAN	HD	HPE	HPR	HA	HAN	HD	HPE	HPR
**Days to 50% Flowering (days)**	−2.09	0.23 **	−8.13	−1.24 *	−0.47 **	−1.46 *	−0.73 **	−7.46	0.84 **	−2.54	−6.03	−6.93	−6.79	−5.42	−4.02	−2.09	−0.71 **	−4.45	2.39 **	0.07 **
**Days to Maturity (days)**	−0.74	0.37 **	−3.5	0.88 **	−0.72	0.02 *	−2.03	−1.79	0.43 **	−1.18	0.18 *	−0.92	−3.99	−0.02 *	0.58 **	0.26 **	−0.82	−3.25	1.6 **	1.1 **
**Plant Height (cm)**	21.08	31.39	17.49	20.69	32.43	37.96 *	53.05 **	49.65 **	19.16	36.8 *	26.63	56.56 **	3.27	16.71	35.5	29.03	32	29.94	31.72	37.25 *
**Head Diameter (cm)**	−7.07	−8.44	−4.31	38.59 **	−4.49	−8.61	−9.33	49.86 **	35.19 **	−7.83	−10.99	−13.96	−4.82	32.47 **	−9.43	−8.46	−11.22	4.2	30.09 **	−0.41
**No. of Leaves/Plant**	−17.59	−6.78	−1.38	2.61	−2.54	−1.27	5.86 **	22.6 **	1.53	0.18	−11.34	−7.46	−2.05	3.03	−7.87	−9.84	0.97	5.55 **	14.54 **	7.48 **
**Specific Leaf Weight (g)**	−7.04 *	−4.74 *	−10.06 *	30.77 **	−35.05	−28.64	−27.78	−67.46	−13.1	−10.0 *	−47.2	−24.44	−37.87	−14.24	−14.11	13.48 **	−46.51	−30.77	−21.71	−8.72 *
**Relative Leaf Water Content (%)**	−3.3 *	−17.48	−17.31	−10.65	−9.69	−10.22	−16.63	8.38 **	−12.24	12.19 **	−2.72 *	4.43 **	−8.59	−4.49	0.72 **	−8.14	−5.83	−14.98	−7.19	−5.9
**SPAD Reading**	−19.62	−0.4 **	−10.41	−2.24 **	4.87 **	−17.09	−18.09	−15.93	−2.21 **	−11.05	−2.59 **	−10.79	−20.44	−4.48 *	8.11 **	−17.81	−12.65	−12.18	−1.64 **	4.22 **
**100-Seed Weight (g)**	3.51 *	3.3 *	−8.42	1.67	−14.42	−4.46	−3.71	4.11 **	10.06 **	−15.55	−13.54	−2.99	−7.85	5.87 **	−2.08	1.91	12.52 **	2.82 *	6.79 **	0.98
**Seed Yield/Plant (g)**	72.49	168.09 **	57.45	80.01	102.74	42.12	199.37 **	35.51	70.03	87.9	20.61	143.18 **	19.63	66.99	77.05	84.91	186.4 **	82.16	77.45	127.28 **
**Harvest Index (%)**	88.11 *	31.13	21.73	29.21	37.34	115.94 **	100.76 **	140.02 **	47.26	98.27 **	16.35	1.25	34.19	3.96	10.58	114.37 **	137.33 **	145.63 **	52.98	108.82 **
**Biochemical Traits**																				
**Oil Content (%)**	7.83 **	7.75 **	−9.15	4.09	8.08 **	6.27 **	7.17 **	−13.26	3.52	2.33	4.79 *	4.8 *	−4.56	3.76	1.66	6.17 **	−3.97	−1.15	4.21	7.92 **
**Palmitic Acid (%)**	−51.58	−46.62	−45.5	−33.62	−39.7	−16.66 **	4.84 **	−15.77 **	−25.57	−23.75	−19.11 **	−21.18 *	−9.17 **	−28.64	−32.28	−37.98	−15.53 **	−14.8 **	−41.37	−52.35
**Stearic Acid (%)**	−38.64	−30.21	−36.71	−19.21 **	−56.63	−27.26	5.33 **	−20.89 **	−35.37	−22.44 **	−27.63	−31.93	−63.69	−32.03	−22.77 **	−29.68	−25.62 *	−56.39	−47.41	−23.04 **
**Oleic Acid (%)**	−19.67	−21.32	−18.92	−25.92	−36.45	−27.28	−36.12	−33.59	−13.0 **	−21.48	−30.59	−23.73	−20.44	−24	−16.87 *	−14.56 **	−8.26 **	−23.44	−6.42 **	−6.78 **
**Linoleic Acid (%)**	26.78 **	30.11 **	21.57	23.4 *	43.76 **	10.78	11.17	16.33	1.94	8.33	40.89 **	31.22 **	35.07 **	25.71 **	12.28	−1.66	−15.8	8.68	−2	−1.39

Note: ** and * indicate significance at *p* < 0.01 and *p* < 0.05, respectively; ^+^ The codes represent sources from HA (*Helianthus argophyllus*), HAN (*Helianthus annuus*), HD (*Helianthus debilis*), HPE (*Helianthus petiolaris*), and HPR (*Helianthus praecox)*.

**Table 2:** Heterosis over better-parent values (BPH, %) among CMS sources representing five different species of sunflower for morphophysiological, yield, and biochemical traits under the water stress environment.

Traits	P100R					P124R					P69R					RCR8297				
	^+^HA	HAN	HD	HPE	HPR	HA	HAN	HD	HPE	HPR	HA	HAN	HD	HPE	HPR	HA	HAN	HD	HPE	HPR
**Days to 50% Flowering (days)**	1.48 **	0.37	1.96 **	6.43 **	−2.55	−1.58	−3.90	0.95 *	0.53	−1.64	−4.80	−5.60	−5.03	−3.37	−3.78	2.53 **	−1.45	3.93 **	4.81 **	−0.80
**Days to Maturity (days)**	0.30 **	−1.44	−3.85	3.13 **	−5.10	−1.31	−1.93	−2.51	0.56 **	−2.83	−1.87	−1.42	−5.19	1.35 **	−2.36	0.92 **	−1.12	−2.18	1.45 **	−2.10
**Plant Height (cm)**	25.52	49.40 **	46.55 **	38.05	42.23 *	6.99	56.06 **	19.47	23.18	23.99	−6.35	74.90 **	6.03	31.15	43.97 *	33.63	55.30 **	34.47	39.54	23.74
**Head Diameter (cm)**	9.38	−16.68	−1.97	46.88 **	−6.48	1.43	−10.35	−3.73	48.36 **	−18.72	−7.73	−5.69	−11.32	31.41 **	−14.28	5.51	−1.52	6.58	43.11 **	−7.86
**No. of Leaves/Plant**	18.83 **	36.03 **	7.26	23.27 **	12.14	14.78	16.52 *	1.01	27.93 **	0.28	−8.09	12.29	−9.29	14.24	5.62	10.59	25.33 **	15.39	18.45 *	−5.01
**Specific Leaf Weight (g)**	−42.54	−30.80	20.83 **	28.62 **	−32.46	−54.16	−41.70	−51.67	−17.31	−15.64 *	−57.04	−43.08	−15.45 *	−25.41	−26.72	−25.74	−42.81	−28.33	−4.75 **	2.50 **
**Relative Leaf Water Content (%)**	−7.64	−2.91	3.91	0.95	14.97 **	5.59	9.78 *	−19.31	22.65 **	27.88 **	−12.82	−7.55	−11.12	2.85	−1.05	4.90	−30.38	27.92 **	6.60	14.82 **
**SPAD Reading**	−15.51	−2.24 **	−23.26	−0.11 **	−17.96	−20.38	−8.69 **	−21.55	−2.53 **	−26.15	−16.33	−11.60	−15.93	−5.04 **	−13.28	−17.66	−18.99	−19.96	−10.45 *	−19.94
**100-Seed Weight (g)**	16.97 *	−5.36	24.53 **	11.11	−12.39	9.04	2.84	12.81	12.12	−9.92	4.27	8.51	16.44 *	16.92 *	0.59	13.37	1.64	44.62 **	24.72 **	−3.59
**Seed Yield/Plant (g)**	63.08 **	16.37	80.48 **	71.75 **	46.56	25.37	62.16 *	46.81	43.91	16.45	28.98	38.82	63.96 **	115.40 **	48.15	41.77	51.98	56.94	28.25	44.38
**Harvest Index (%)**	−35.84	−36.33	−23.87 **	−43.57	−48.46	−45.78	−26.36 **	−40.64	−51.17	−44.43	−51.07	−42.34	−11.34 **	−46.09	−48.04	−17.51 **	−12.50 **	−4.67 **	−42.67	−24.74 **
**Biochemical Traits**																				
**Oil Content (%)**	13.64 **	15.07 **	8.58	4.21	4.51	8.70	8.28	4.74	7.23	2.91	9.25	9.50 *	10.71 **	10.26 **	4.67	11.73 **	5.61	1.24	10.86 **	5.96
**Palmitic Acid (%)**	−14.41	−18.92	−19.85	−10.74 *	−34.58	−0.92 **	−49.71	−66.37	−13.63 *	−1.36 **	2.43 **	−15.36	−60.44	−11.43 *	4.84 **	−37.48	−25.19	−18.07	−20.06	−19.43
**Stearic Acid (%)**	−64.72	−39.11 *	−70.31	−54.05	−75.42	−42.41 *	0.74 **	−53.38	−34.42 **	−34.88 **	−27.20 **	−55.12	−54.69	−41.47 *	−26.70 **	−44.90 *	−44.57 *	−84.17	−53.17	−45.85 *
**Oleic Acid (%)**	1.20 **	−15.21	−21.61	1.99 **	−13.56	−7.43 *	−16.40	−27.13	−8.98 *	−14.85	−30.09	−16.14	−30.78	−14.58	−31.15	−3.91 **	−10.90	−29.65	0.50 **	6.60 **
**Linoleic Acid (%)**	1.16	5.11	24.98 **	−3.77	12.00	−15.20	−8.85	4.15	−7.04	1.32	30.59 **	14.32 *	56.31 **	8.00	19.59 **	−10.75	−11.26	13.14 *	−10.32	−13.74

Note: ** and * indicate significance at *p* < 0.01 and *p* < 0.05, respectively. ^+^ The codes represent sources from HA (*H. argophyllus*), HAN (*H. annuus*), HD (*H. debilis*), HPE (*H. petiolaris*), and HPR (*H. praecox)*.

**Table 3:** Heterosis over mid-parent values (MPH, %) among CMS sources representing five different species of sunflower for morphophysiological, yield, and biochemical traits under normal irrigation.

Traits	P100R					P124R					P69R					RCR8297				
	^+^HA	HAN	HD	HPE	HPR	HA	HAN	HD	HPE	HPR	HA	HAN	HD	HPE	HPR	HA	HAN	HD	HPE	HPR
**Days to 50% Flowering (days)**	−1.82	0.65 **	−5.5	−0.47 *	0.64 **	−1.15	−0.54 *	−4.26	1.2 **	−1.47	−3.19	−3.98	−6.69	−2.08	−1.69	−1.55	−0.3 *	−0.92	2.76 **	1.18 **
**Days to Maturity (days)**	−0.05	1.1	−1.82	1.67 *	0.24	1.36	−0.39	0.84	1.72 *	0.62	1.7 *	0.9	−1.26	1.44	2.57 **	2.12 **	1.34	−0.17	3.43 **	3.48 **
**Plant Height (cm)**	36.07	54.07 **	24.6	29.81	43.15	48.01	60.53 **	59.24 **	42.64	46.97	45.22	68.78 **	23.49	54.24 **	60.07 **	44.26	56.75 **	39.82	39.64	47.79
**Head Diameter (cm)**	14.8	14.43	14.9	45.95 **	17.55	13.37	13.6	80.5 **	42.5 **	13.5	11.47	8.6	15.78	41.6 **	13.05	24.3	21.53	38.14 **	54.17 **	34.49 *
**No. of Leaves/Plant**	−11.6	8.05	4.23	10.39	3.94	1.44	18.26 **	24.29 **	10.81	6.35	−0.66	11.95	8.63	9.11	−2.08	−1.32	19.35 **	13.93 **	21.69 **	14.67 **
**Specific Leaf Weight (g)**	12.07 **	22.85 **	18.75 **	45.27 **	−19.79	−23.44	−20.41	−62.71	−7.11	−4.67	−42.2	−16.62	−31.37	−4.39	−10.78	51.12 **	−23.66	0.86	−0.85	26.14 **
**Relative Leaf Water Content (%)**	−1.78	−12.24	−12.46	−4.54	−3.45	−1.17	−7.81	10.86 **	−8.12	15.04 **	1.8 *	10.85 **	−5.59	0.03	4.72 **	−1.77	1.92 *	−14.31	−4.17	−3.14
**SPAD Reading**	−12.26	8.98 **	−1.53	5.34 **	12.58 **	−11.3	−12.15	−9.51	2.97 **	−6.58	0.01	−8.34	−18.05	−1.7	9.26 **	−15.18	−10.17	−9.96	3.1 **	9.73 **
**100-Seed Weight (g)**	6.23	6.81	−4.12	4.73	−5.45	−0.93	−0.56	5.56	14.85 **	−6.88	−9.53	3.77	−0.28	10.67 **	8.23 *	9.79 **	18.75 **	7.24	17.25 **	11.03 **
**Seed Yield/Plant (g)**	111.65	185.94 **	100.77	117.99	127.24	87.98	213.82 **	85.37	120.8	129.45	54.15	156.35 *	59.24	113.04	109.02	155.55 *	220.1 **	161.37 **	144.04	193.99 **
**Harvest Index (%)**	131.55 *	86.37	48	45.16	79.42	145.09 **	164.96 **	165.51 **	71.07	159.01 **	51.48	54.08	78.52	20.59	40.12	149.31 **	226.51 **	185.07 **	74.05	161.89 **
**Biochemical Traits**																				
**Oil Content (%)**	11.0 **	9.31 *	−3.91	7.19	13.2 **	10.51 **	10.04 **	−5.5	7.4	7.06	8.72	6.92	3.27	7.09	6.4	10.31 **	−1.66	7.33	7.78	12.71 **
**Palmitic Acid (%)**	−41.84	−35.13	−33.74	−24.61	−25.42	−5.86 **	13.0 **	−9.08 **	−20.45	−8.23 **	−8.45 **	−16.04	−3.18 **	−22.64	−18.25	−30.99	−12.61 *	−11.78 *	−35.23	−42.99
**Stearic Acid (%)**	−32.67	−15.7 *	−32.9	−11.81 **	−52.1	−20.54	24.87 **	−8.24 **	−30.05	−15.82 *	−20.8	−15.01 *	−61.63	−25.11	−14.26 *	−22.46	−3.79 **	−54.16	−41.36	−15.86 *
**Oleic Acid (%)**	−15.6	−19.05	−15.06	−20.77	−29.84	−19.45	−26.92	−26.41	−5.81 **	−14.47	−28.9	−21.33	−20.36	−20.21	−8.37 **	−4.02 **	5.58 **	−14.27	1.81 **	0.56 **
**Linoleic Acid (%)**	38.52 **	40.08 **	34.23 *	37.56 **	59.3 **	25.02	27.02	30.21	11.95	18.33	43.48 **	36.07 **	35.35 **	32.63	20.66	11.44	−2.73	22.35	8.34	9.04

Note: ** and * indicate significance at *p* < 0.01 and *p* < 0.05, respectively. ^+^ The codes represent sources from HA (*H. argophyllus*), HAN (*H. annuus*), HD (*H. debilis*), HPE (*H. petiolaris*), and HPR (*H. praecox)*.

**Table 4:** Heterosis over mid-parent values (MPH, %) among CMS sources representing five different species of sunflower for morphophysiological, yield, and biochemical traits under the water stress environment.

Traits	P100R					P124R					P69R					RCR8297				
	^+^HA	HAN	HD	HPE	HPR	HA	HAN	HD	HPE	HPR	HA	HAN	HD	HPE	HPR	HA	HAN	HD	HPE	HPR
**Days to 50% Flowering (days)**	2.29 *	1.16	2.08	7.02 **	−0.6	−0.09	−2.81	2.77 **	2.66 **	−0.24	−1.8	−3	−1.77	0.25	−2.3	3.44 **	−0.18	4.56 **	5.38 **	1.63
**Days to Maturity (days)**	1.38 *	0.38	−1.96	4.02 **	−2.83	0.29	0.39	−0.08	1.77 **	−0.01	−0.03	1.17	−2.58	2.85 **	0.74	2.28 **	0.93	0.0	2.42 **	0.49
**Plant Height (cm)**	41.79	70.61 **	50.35	60.45 *	59.44 *	27.37	77.06 **	29.98	50.47	37.58	16.41	94.22 **	21.15	67.27 **	59.04 *	36.63	78.31 **	45.46	49.67	38.56
**Head Diameter (cm)**	46.51 **	19.47	31.93	68.22 **	30.25	31.63	23.75	24.67	62.75 **	9.05	14.47	24.82	9.59	43.72 *	10.4	45.19 **	42.79 *	46.51 **	68.59 **	30.49
**No. of Leaves/Plant**	23.37	43.93 **	10.81	42.79 **	20.18	20.42	33.3 **	6.64	36.86 **	8.86	2.97	36.49 **	2.37	21.12	14.15	16.99	44.47 **	22.89	25.34	3.29
**Specific Leaf Weight (g)**	−25.17	−4.2	42.86 **	31.67 **	−18.8	−45	−24.89	−48.21	−9.64	−7.33	−52.05	−29.76	−14.4	−12.34	−22.88	−3.17 *	−21.24	−16.1	−3.2 *	23.02 **
**Relative Leaf Water Content (%)**	−4.78	6.78	12.52	8.99	26.49 **	18.2 *	20.94 **	−19.22	26.63 **	29.93 **	−8.82	1.17	−5.13	9.5	7.27	10.18	−23.44	36.02 **	13.62	24.05 **
**SPAD Reading**	−9.17	0.02 **	−16.88	4.8 **	−10.49	−5.67	2.9 **	−6.34	10.38 **	−11.25	−13.32	−8	−13.71	−0.27 **	−10.33	−13.61	−10.22	−16.62	−2.08 **	−17.22
**100-Seed Weight (g)**	22.79 *	−0.97	30.5 **	16.91	−1.05	16.35	13.24	15.73	17.7	0.95	10.57	17.02	16.57	22.82 *	13.55	21.51	13.5	50.48 **	32.74 **	7.38
**Seed Yield/Plant (g)**	82.8	29.4	95.73 **	106.74 **	59.41	39.04	79.66	56.23	70.61	28.55	60.65	78.63	104.83 **	158.73 **	107.02 **	60.31	66.37	80.98	59.63	47.87
**Harvest Index (%)**	−9.38	−4.86	9.88 **	−18.28	−24.67	−17.03	18.34 **	−8.22	−23.81	−20.69	−33	−14.68	25.42 **	−21.22	−24.49	13.62 **	34.9 **	37.64 **	−15.7	9.36 **
**Biochemical Traits**																				
**Oil Content (%)**	15.96	15.5	9.08	6.79	8.29	14.03	13.76	10.94	11.92	8.22	56.39	56.94 **	59.43 **	57.46 **	49.84 **	16.67	10.49	6.77	15.18	10.88
**Palmitic Acid (%)**	−7.27	−7.36	−6.32	−1.46 *	−28.48	9.43 **	−43.01	−61.98	−8.04	8.33 **	16.2	−1.32 *	−58.08	−8.42	16.11 **	−25.83	−11.09	−17.09	−15.74	−4.53
**Stearic Acid (%)**	−58.58	−26.83 *	−67.75	−39.66	−67.16	−32.56	19.64 **	−35.11	−26.97 *	−33.8	−22.62	−45.93	−40.12	−30.94	−19.95 **	−40.98	−34.7	−81.63	−42.92	−31.32
**Oleic Acid (%)**	4.35 **	−8.48	−17.45	4.9 **	−5.84	5.67 **	−2.48	−15.29	0.92 *	−8.12	−27.33	−11.27	−29.55	−8.32	−25.52	10.27 **	4.76 **	−17.75	12.22 **	15.57 **
**Linoleic Acid (%)**	6.03	11.78	39.53 **	0.73	20.46	−2.33	7.9	29.6 **	3.06	11.05	38.46	23.08	63.98 **	16.87	31.33 **	0.19	2.36	37.17 **	−3.92	−6.1

Note: ** and * indicate significance at *p* < 0.01 and *p* < 0.05, respectively. ^+^ The codes represent sources from HA (*H. argophyllus*), HAN (*H. annuus*), HD (*H. debilis*), HPE (*H. petiolaris*), and HPR (*H. praecox)*.

**Table 5:** Heterosis over commercial check values (%) among CMS sources representing five different species of sunflower for morpho-physiological, yield and biochemical traits under normal irrigation.

Traits	P100R					P124R					P69R					RCR8297				
	^+^HA	HAN	HD	HPE	HPR	HA	HAN	HD	HPE	HPR	HA	HAN	HD	HPE	HPR	HA	HAN	HD	HPE	HPR
Days to 50% Flowering (days)	95.22	97.47 **	94.61	96.04	98.16 **	95.29	95.75	95.29	97.07 *	95.53	96.56	95.64	95.98	97.19 *	98.62 **	94.68	95.75	98.39 **	98.33 **	97.87 **
Days to Maturity (days)	95.68	97.11 **	95.25	97.27 **	96.42	96.14	94.81	96.94 **	96.42	95.9	96.3	95.86	94.77	96	97.59 **	96.35	95.94	95.5	97.55 **	98.15 **
Plant Height (cm)	84.11	90.17	80.63	96.95 **	91.89 *	80.62	81.84	90.98 *	95.65 **	83.25	71.2	75.21	62.78	93.62 **	81.19	91.64 *	93.46 **	92.0 *	105.82 **	97.17 **
Head Diameter (cm)	95.89	96.99	91.09	97.88	98.61	94.61	95.97	142.67 **	94.93	94.56	92.18	90.61	90.61	93.48	93.95	94.54	93.63	99.2	91.84	102.29 *
No. of Leaves/Plant	56.47	63.88	67.58	80.69 **	70.67	62.07	66.55	77.07 **	77.45 **	69.63	67.49	70.45	74.57	83.84 **	70.93	64.65	72.4	75.69 *	90.83 **	80.19 **
Specific Leaf Weight (g)	86.3 *	109.93 **	104.11 **	97.43 **	62.67	68.04	80.48	37.67	75	87.67 *	53.65	92.47 **	71.92	80.48	85.62	100.46 **	61.64	80.14	58.39	88.36 *
Relative Leaf Water Content (%)	107.68 **	98.25	89.2	96.38	97.43	100.76	95.47	103.95 *	85.21	106.29 **	109.39 **	121.59 **	93.62	98.39	103.15 *	102.91	109.37 **	82.85	92.22	93.19
SPAD Reading	82.35	102.56 **	93.1	97.46	103.53 **	85.13	84.54	87.36	97.13	87.74	100.34 **	92.32	82.68	97.04	107.28 **	89.84	95.48	96	107.51 **	113.92 **
100-Seed Weight (g)	86.75 **	84.94	75.3	87.42 **	77.41	77.81	76.66	80.12	92.73 **	71.46	76.31	85.62 *	81.33	95.29 **	91.11 **	81.93	86.52 *	76.96	89.61 **	82.76
Seed Yield/Plant (g)	105.76 **	101.11	104.47 **	106.84 **	97.77	87.45	99.87	89.91	98.68	90.6	73.27	85.75	79.37	101.01	85.29	111.04 **	95.44	120.86 **	104.79 **	109.11 **
Harvest Index (%)	232.87 **	162.33	150.7	208.71	181.99	217.65	198.39	237.19 **	218.94	226.21 *	184.57	160.61	212.86	194.48	175.4	236.66 **	262 **	271.17 **	236.15 **	272.56 **
Biochemical Traits																				
Oil Content (%)	104.61 **	103.52 **	97.03	102.76 *	108.49 **	100.88	100.95	92.64	99.8	99.35	99.85	98.92	101.94	100.31	99.48	101.17	90.66	105.58 **	100.57	104.65 **
Palmitic Acid (%)	80.37	88.6	90.47	110.19 **	100.09	109.47 **	131.68 **	105.79 **	98.69	103.27 *	104.86 *	96.36	111.03 **	94.72	89.81	76.57	97.1	97.94	75.79	61.68
Stearic Acid (%)	103.59	105.9	108.33	130.56 **	69.1	107.06	130.56 **	135.42 **	94.44	109.9	122.34 **	103.99	62.15	110.85	122.74 **	116.67 *	114.93 *	74.65	90.36	125.17 **
Oleic Acid (%)	101.8 **	101.19 **	102.33 **	93.51	82	83.63	79.01	76.21	95.38	86.86	82.1	93.15	91.29	89.35	101.14 **	99.1 **	112.37 **	87.86	101.29 **	100.04 **
Linoleic Acid (%)	100.28	99.8	98.35	103.08	118.84 **	113.92 **	114.32 **	119.63 **	104.83	111.4 **	114.96 **	106.61	109.27	109.97 *	98.67	102.46	87.73	113.23 **	102.11	102.75

Note: ** and * indicate significance at *p* < 0.01 and *p* < 0.05, respectively. ^+^ The codes represent sources from HA (*H. argophyllus*), HAN (*H. annuus*), HD (*H. debilis*), HPE (*H. petiolaris*), and HPR (*H. praecox*).

**Table 6:** Heterosis over commercial check values (%) among CMS sources representing five different species of sunflower for morphophysiological, yield, and biochemical traits under water stress environment.

Traits	P100R					P124R					P69R					RCR8297				
	^+^HA	HAN	HD	HPE	HPR	HA	HAN	HD	HPE	HPR	HA	HAN	HD	HPE	HPR	HA	HAN	HD	HPE	HPR
**Days to 50% Flowering (days)**	97.43	96.73	96.97	101.34 **	96.27	97.04	94.76	99.54 **	99.13 **	98.49 *	96.97	96.15	96.74	98.43 *	98.01	98.06	94.99	98.84 **	99.3 **	97.9
**Days to Maturity (days)**	98.22 *	98	95.83	100.38 **	95.42	96.67	97.5	97.17	97.71	97.67	96.11	98	94.5	98.5 **	98.17 *	98.83 **	98.25 *	97.5	98.59 **	98.42 **
**Plant Height (cm)**	89.18	95.83 *	84.49	105.7 **	89.2	76.04	94.39 *	68.87	94.24 *	72	66.53	97.25 **	61.13	100.78 **	79.41	94.92 *	110.96 **	91.32	108.99 **	86.19
**Head Diameter (cm)**	113.62 **	108.33	101.19	105.69	108.57	107.79	114.7 **	99.37	106.4	93.88	98.39	120.26 **	91.53	103.5	99.76	111.94 **	124.67 **	110.01 *	102.99	105.84
**No. of Leaves/Plant**	76.27	80.8	68.07	101.62 **	77.84	81.44	82.67	71.67	105.2 **	77.18	75.53	92.28 **	74.55	100.71 **	87.4	79.94	90.59 **	83.41	96.92 **	73.2
**Specific Leaf Weight (g)**	62.13	93.54 **	98.64 **	76.02 *	57.82	49.66	79.25 **	39.46	58.5	73.13	46.26	81.63 **	70.75	62.41	66.33	81.86 **	77.89 *	58.5	56.63	89.46 **
**Relative Leaf Water Content (%)**	92.91	105.35 **	98.18	95.38	108.63 **	107.27 **	109.88 **	64.57	101.84 *	102.09 *	87.6	97.24	81.4	94.2	90.63	105.48 **	74.29	116.21 **	97.02	104.31 **
**SPAD Reading**	93.94	97.2	86.52	103.04 **	94.01	88.33	90.03	88.45	97.52	84.68	94.43	94.52	94.78	103.75 **	99.28 *	100.92 **	99.29 *	98.1	109.75 **	98.13
**100-Seed Weight (g)**	105.77 **	90.88	110.31 **	101.34 *	87.11	93.19	96.78	90.79	95.03	82.23	90.85	102.95 **	93.92	101.52 *	96.41	96.13	95.86	116.39 **	106.08 **	85.64
**Seed Yield/Plant (g)**	131.57 **	95.96	138.3 **	131.61 **	134.11 **	101.06	132.2 **	107.99	106.42	105.7	86.53	100.3	106.06	118.92	133.81 **	126.68 *	136.55 **	138.24 **	112.97	132.16 **
**Harvest Index (%)**	203.3	201.74	241.24	178.82	190.39	225.77	306.63 **	247.19 *	203.31	240.06	144.53	170.34	261.91 **	159.24	186.74	261.84 **	277.75 **	302.58 **	181.97	280.11 **
**Biochemical Traits**																				
**Oil Content (%)**	118.93 **	118.52 **	112.88 *	109.01	111.47	110.85	110.63	108.88	108.22	105.65	111.39	111.92	115.09 **	111.5	106.94	113.9 **	107.94	105.24	111.84	108.65
**Palmitic Acid (%)**	82.43	78.15	97.3 **	95.37 *	62.5	97.48 **	50.54	40.83	92.13	96.13 *	111.63 **	91.19	48.02	98.11 **	112.95 **	74.1	88.67	99.46 **	95.73 *	95.5 *
**Stearic Acid (%)**	116.74	201.5 **	116.74	152.04	81.33	141.2	192.7 **	183.26 **	122.64	111.8	168.1 **	103.43	178.11 **	125.32	147.85	156.8 *	157.73 *	62.23	133.26	154.08
**Oleic Acid (%)**	96.42 **	89.42 **	77.89	93.81 **	83.27	88.31 *	84.43	72.41	81.24	72.62	72.36	91.49 **	71.27	87.95 *	71.58	91.52 **	90.41 **	69.9	89.85 **	90.56 **
**Linoleic Acid (%)**	106.03	110.72	127.77 *	105.57	127.72 *	112.97	121.45	138.76 **	123.85	134.99 **	129.94 **	111.94	139.52 **	114.26	131.94 **	111.17	110.53	140.91 **	111.69	109.61

Note: ** and * indicate significance at *p* < 0.01 and *p* < 0.05, respectively. ^+^ The codes represent sources from HA (*H. argophyllus*), HAN (*H. annuus*), HD (*H. debilis*), HPE (*H. petiolaris*), and HPR (*H. praecox)*.

**Table 7:** Pearson’s correlation coefficients of sunflower hybrids under the regular growth regime with only significant values at *p* < 0.05 (*) and *p* < 0.01 (**).

	Days to Maturity (days)	Plant Height (cm)	No. of Leaves/Plant	Photosynthetic Efficiency (SPAD Reading)	Seed Yield/Plant (g)	100-Seed Weight (g)	Oil Content (%)	Oleic Acid (%)
Days to 50% Flowering (days)	0.41 *		0.37 *	0.41 *				0.33 *
Days to Maturity (days)		0..39 *	0.46 **	0.40 *				
Plant Height (cm)			0.67 **	0.38 *	0.44 **	0.41 *		0.29 *
No. of Leaves/Plant				0.53 *		0.43 *		
Specific Leaf Weight (g)							0.32 *	
100-Seed Weight (g)								0.34 *

**Table 8:** Pearson’s correlation coefficients of sunflower hybrids under water stress with only significant values at *p* < 0.05 (*) and *p* < 0.01 (**).

	Days to Maturity (days)	Plant Height (cm)	Head Diameter (cm)	No. of Leaves/Plant	Photosynthetic Efficiency (SPAD Reading)
Days to 50% Flowering (days)	0.46 **	0.31 *		0.44 **	0.44 **
Days to Maturity (days)		0.50 **		0.35 *	0.39 *
Plant Height (cm)			0.30 *	0.62 **	0.64 **
No. of Leaves/Plant					0.55 **

**Table 9:** The list of genotypes and their interspecific hybrids (*n* = 48) used in the study as adapted from Tyagi et al. [5].

A/B/R Lines	Species		Hybrids			
		Cytoplasm Code	RCR8297	P69R	P124R	P100R
*A Lines (Alloplasmic)*						
CMS-E002-91A	*H. annuus*	HAN	CMS-E002-91A × RCR8297	CMS-E002-91A × P69R	CMS-E002-91A × P124R	CMS-E002-91A × P100R
CMS-PKU-2A	*H. annuus*	HAN	CMS-PKU-2A × RCR8297	CMS-PKU-2A × P69R	CMS-PKU-2A × P124R	CMS-PKU-2A × P100R
CMS-ARG-2A	*H. argophyllus*	HA	ARG-2A × RCR8297	CMS-ARG-2A × P69R	CMS-ARG-2A × P124R	CMS-ARG-2A × P100R
CMS-ARG-3A	*H. argophyllus*	HA	CMS-ARG-3A × RCR8297	CMS-ARG-3A × P69R	CMS-ARG-3A × P124R	CMS-ARG-3A × P100R
CMS-ARG-6A	*H. argophyllus*	HA	CMS-ARG-6A × RCR8297	CMS-ARG-6A × P69R	CMS-ARG-6A × P124R	CMS-ARG-6A × P100R
CMS-DV-10A	*H. debilis* ssp. *Vestitus*	HD	CMS-DV-10A × RCR8297	CMS-DV-10A × P69R	CMS-DV-10A × P124R	CMS-DV-10A × P100R
CMS-PHIR-27A	*H. praecox* ssp. *Hirtus*	HPR	CMS-PHIR-27A × RCR8297	CMS-PHIR-27A × P69R	CMS-PHIR-27A × P124R	CMS-PHIR-27A × P100R
CMS-PRUN-29A	*H. praecox* ssp. *Runyonii*	HPR	CMS-PRUN-29A × RCR8297	CMS-PRUN-29A × P69R	CMS-PRUN-29A × P124R	CMS-PRUN-29A × P100R
*A Lines (Euplasmic lines)*						
CMS-40A	*H. petiolaris ^a^*	HPE	CMS-40A × RCR8297	CMS-40A × P69R	CMS-40A × P124R	CMS-40A × P100R
CMS-42A	*H. petiolaris*	HPE	CMS-42A × RCR8297	CMS-42A × P69R	CMS-42A × P124R	CMS-42A × P100R
CMS-234A	*H. petiolaris*	HPE	CMS-234A × RCR8297	CMS-234A × P69R	CMS-234A × P124R	CMS-234A × P100R
CMS-38A	*H. petiolaris*	HPE	CMS-38A × RCR8297	CMS-38A × P69R	CMS-38A × P124R	CMS-38A × P100R
*R Lines (Restorer Lines)*						
RCR-8297	*H. annuus*	HAN				
P69R	*H. annuus*	HAN				
P124R	*H. annuus*	HAN				
P100R	*H. annuus*	HAN				

*^a^ H. petiolaris* is a conventional source. A line: CMS line, B line: Maintainer of A line, and R line: Restorer line.

## Supplementary Materials

The following are available online at https://www.mdpi.com/2223-7747/9/4/465/s1, Table S1: Data of specific combining ability of sixty one-way F_1_ hybrids under normal and water stress environments. Figure S1. Average performance of sunflower hybrids as better-parent heterosis (BPH, %) developed across groups for yield and oil content. The codes (x-axis) represent sources from HA (*H. argophyllus*), HAN (*H. annuus*), HD (*H. debilis*), HPE (*H. petiolaris*), and HPR (*H. praecox*). The normal (N) and stress (S) environments are represented along the x-axis. Figure S2. Average performance of sunflower hybrids as mid-parent heterosis (MPH, %) developed across groups for yield and oil content. The codes (x-axis) represent sources from HA (*H. argophyllus*), HAN (*H. annuus*), HD (*H. debilis*), HPE (H. petiolaris), and HPR (*H. praecox*). The normal (N) and stress (S) environments are represented along the x-axis. Figure S3. Average performance of sunflower hybrids as heterosis percent of check developed across groups for yield and oil content. The codes (x-axis) represent sources from HA (*H. argophyllus*), HAN (*H. annuus*), HD (*H. debilis*), HPE (*H. petiolaris*), and HPR (*H. praecox)*. The normal (N) and stress (S) environments are represented along the x-axis. Table S1: Mean performance of parental lines of sunflower for morphophysiological, yield, and biochemical traits under normal irrigation pooled over years. Table S2: Mean performance of sunflower hybrids for morphophysiological, yield, and biochemical traits under normal irrigation pooled over years. Table S3: Heterosis over better-parent values (BPH, %) of sunflower for morphophysiological, yield, and biochemical traits under normal irrigation pooled over years. Table S4. Heterosis over mid-parent values (MPH, %) of sunflower for morphophysiological, yield, and biochemical traits under normal irrigation pooled over years. Table S5. Heterosis over commercial check values (%) for morphophysiological, yield, and biochemical traits under normal irrigation pooled over years. Table S6. Weather data during crop season over years used in the study.
