# Supplementary material for: Heterotic Effect of Different Cytoplasmic Combinations in Sunflower Hybrids Cultivated Under Diverse Irrigation Regimes
Source: Plants (Basel). 2020 Apr 7;9(4):465. doi: 10.3390/plants9040465 (PMC7238153; doi:10.3390/plants9040465)
Supplement: Supplementary file 1 [file plants-09-00465-s001.zip › Supplemetary_FiguresS1, S2, and S3.docx]

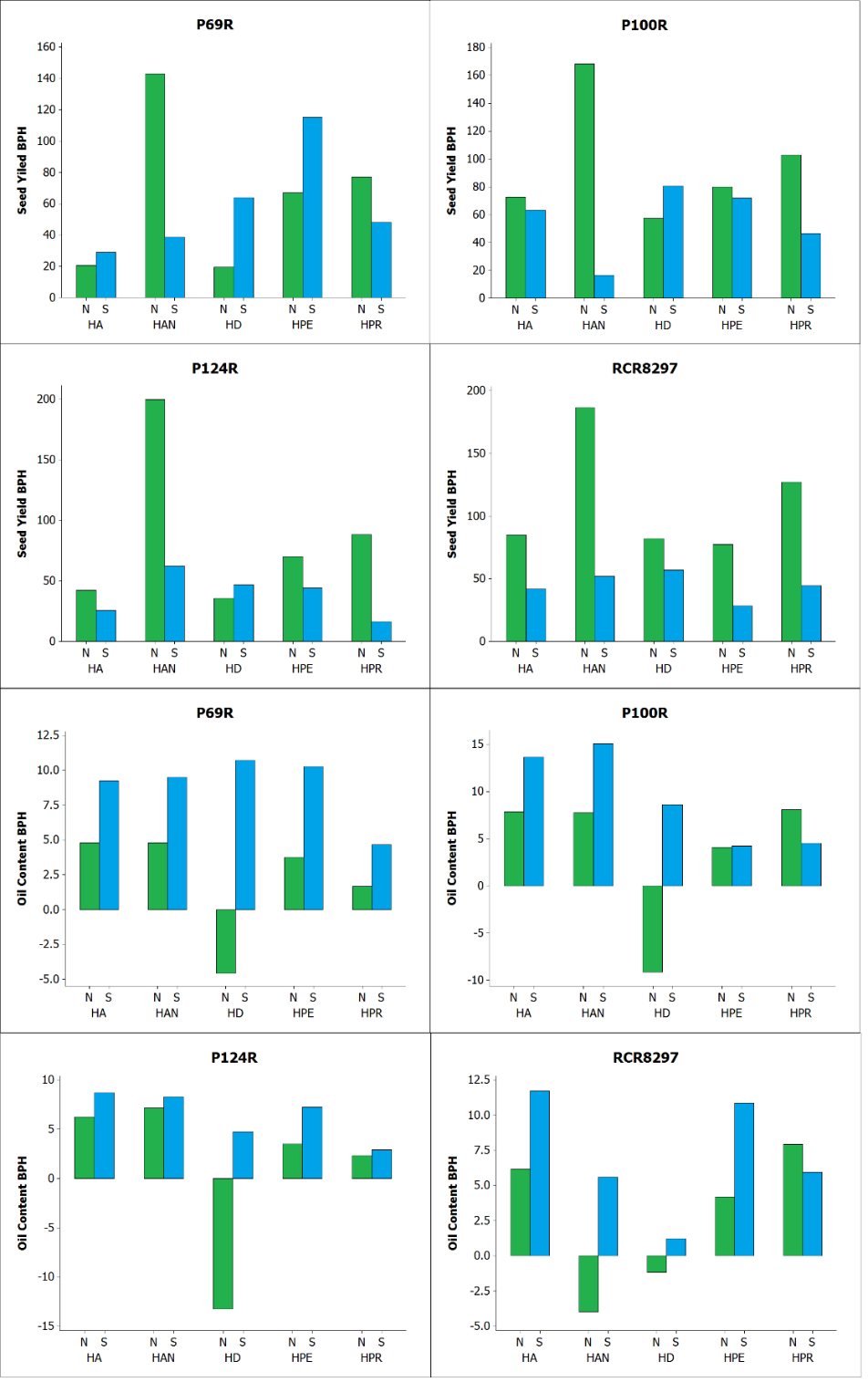


**Figure S1.** Average performance of sunflower hybrids as better parent heterosis (BPH,%) developed across groups for yield and oil content. The codes (x-axis) represent sources from HA (*H. argophyllus*), HAN (*H. annuus*), HD (*H. debilis*), HPE (*H. petiolaris*), and HPR (*H. praecox)*, respectively. The normal (N) and stress (S) environment are represented along the *x-axis*.


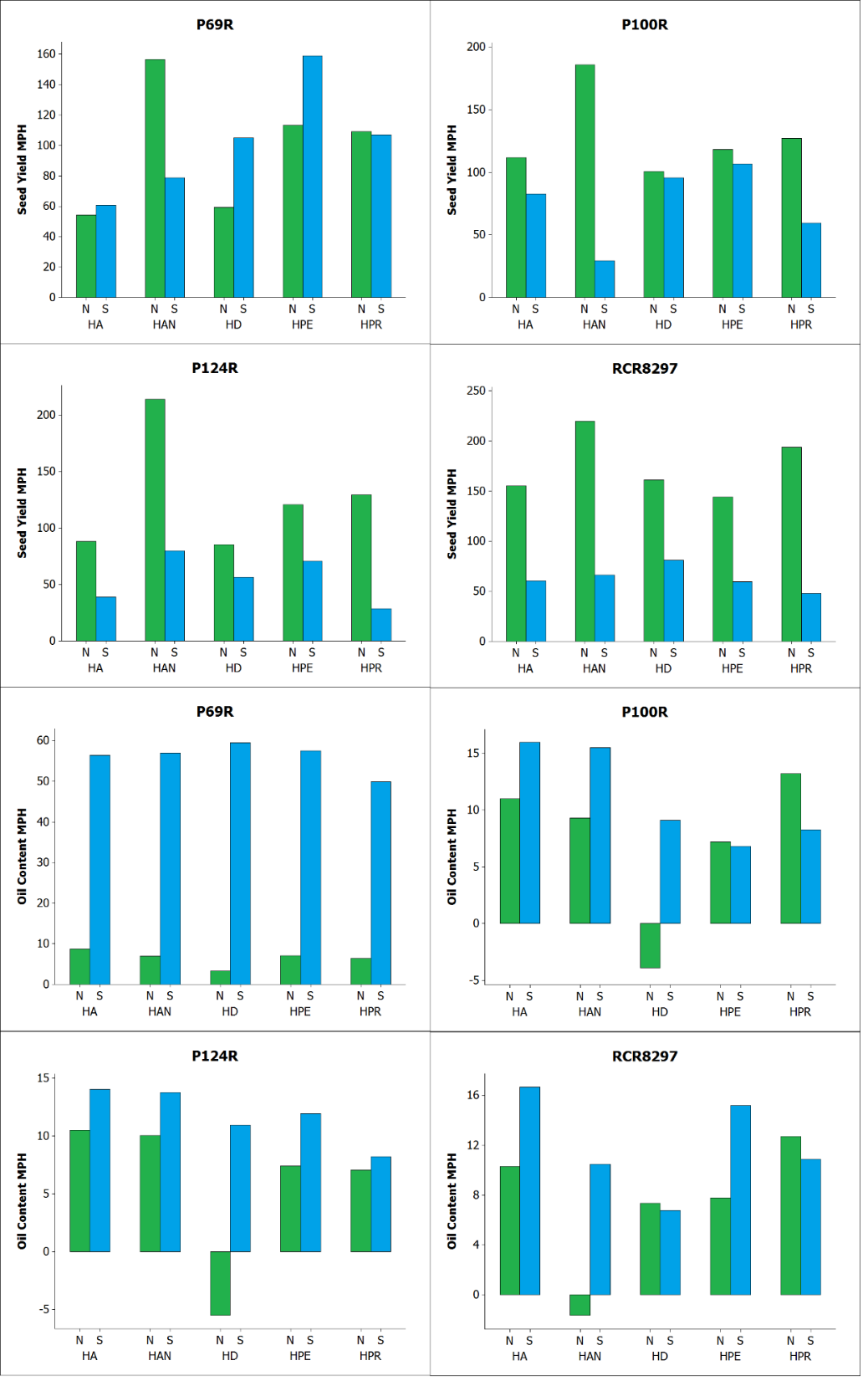


**Figure S2.** Average performance of sunflower hybrids as mid parent heterosis (MPH,%) developed across groups for yield and oil content. The codes (x-axis) represent sources from HA (*H. argophyllus*), HAN (*H. annuus*), HD (*H. debilis*), HPE (*H. petiolaris*), and HPR (*H. praecox)*, respectively. The normal (N) and stress (S) environment are represented along the *x-axis*.


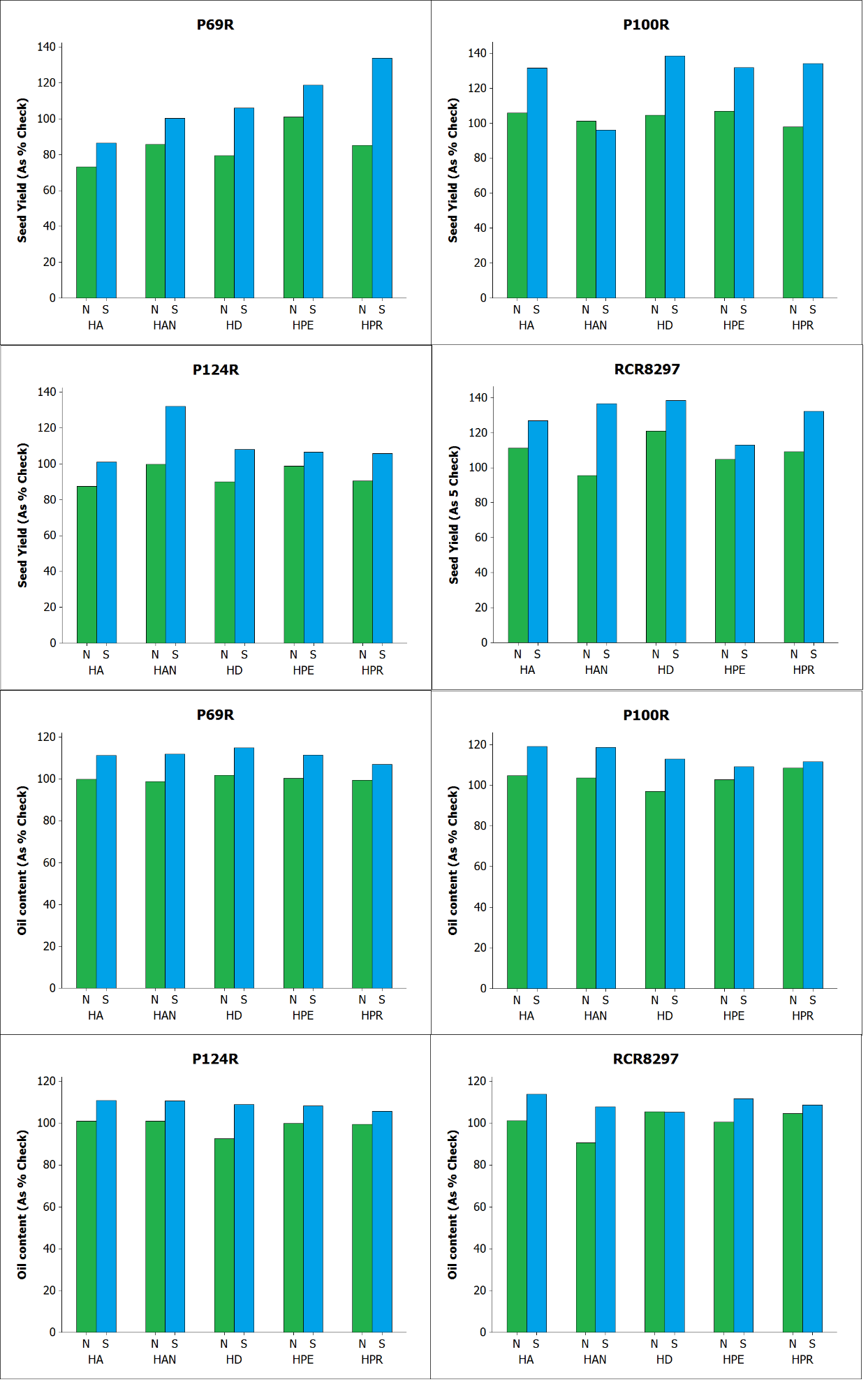


**Figure 3.** Average performance of sunflower hybrids as heterosis as percent of check developed across groups for yield and oil content. The codes (x-axis) represent sources from HA (*H. argophyllus*), HAN (*H. annuus*), HD (*H. debilis*), HPE (*H. petiolaris*), and HPR (*H. praecox)*, respectively. The normal (N) and stress (S) environment are represented along the *x-axis*.
